# Effects of Thyme, Cumin, and Sumac Extracts on Apoptosis and Paraptosis in Hepatocellular Carcinoma: Synergistic, Antagonistic, or Additive Properties

**DOI:** 10.1002/fsn3.70106

**Published:** 2025-03-24

**Authors:** Yagmur Yasar Firat, Betul Cicek, Ayca Kara, Nurefsan Konyaligil Ozturk, Selen Ilgun

**Affiliations:** ^1^ Department of Nutrition Dietetic, Faculty of Health Sciences Erciyes University Kayseri Türkiye; ^2^ Betül Ziya Eren Genom and Stem Cell Center Erciyes University Kayseri Türkiye; ^3^ Department of Nutrition Dietetic, Faculty of Health Sciences Bolu Abant İzzet Baysal University Bolu Türkiye; ^4^ Department of Pharmacognosy, Faculty of Pharmacy Erciyes University Kayseri Türkiye

**Keywords:** apoptosis, cumin, HepG2, paraptosis, sumac, thyme

## Abstract

This study evaluated the effect of single, double, and triple combined doses of sumac, thyme, and cumin extracts on apoptosis and paraptosis in the HepG2 cell line. The effect of thyme and cumin extracts was higher in proteins (mTOR, caspase‐8, caspase‐9, Bax and bcl‐2) other than caspase‐3 protein. The expression of caspase‐3 protein was higher in the sumac extract‐treated groups. The expression levels of GRP78/Bip and DDIT3/Chop proteins, which are indicators of paraptosis, did not exert a significant difference between the extracts. Even though their protein expression is different, according to MTT results, sumac and thyme extracts showed an additive effect, thyme and cumin extracts showed an antagonistic effect, sumac and cumin extracts showed a synergistic effect, and sumac, thyme, and cumin extracts showed a synergistic effect. Sumac, thyme, and cumin extracts induced cell death by causing apoptosis in HepG2 cells, and they may have a supportive impact on the treatment of hepatocellular carcinoma.

## Graphical abstract

While sumac induced apoptosis via caspase‐3 expression, thyme and cumin caused apoptosis by altering the expression of caspase‐8, caspase‐9, bax, and bcl‐2 proteins. In cell cytotoxicity, sumac and thyme showed an additive effect, thyme and cumin showed an antagonistic effect, and sumac and cumin showed a synergistic effect.

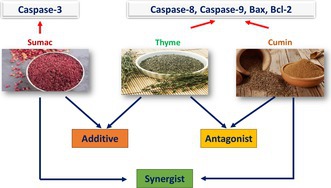

## 1 Introduction

Hepatocellular carcinoma (HCC), hepatocarcinoma, or hepatoma accounts for 75%–85% of all primary liver cancer cases. It is a tumor formed in hepatocytes, liver epithelial cells (Townsend Jr. et al. 2015). It ranks sixth among the most common cancers in the world and third in cancer‐related deaths (Sung et al. 2021). Many pathways, such as MAPK (Moon and Ro 2021), PI3K‐Akt–mTOR (Sun et al. 2021), Wnt‐β‐catenin (Vilchez et al. 2016), proangiogenic, and epidermal growth factor receptor pathway (Prenner and Kulik 2016) are involved in the pathogenesis of HCC. Since these pathways are generally related to cell proliferation, cell adhesion, apoptosis, and angiogenesis, cell proliferation and apoptosis play an important role in HCC development and progression (Prenner and Kulik 2016).

Apoptosis is a homeostatic mechanism that maintains cell populations during tissue development and aging. It also appears as a defense mechanism in immune reactions or when cells are damaged by disease or harmful agents (Elmore 2007). Apoptosis represents a physiological way of eliminating excess cells during liver development and regeneration. Therefore, inadequate apoptosis has been associated with the development and progression of liver and bile duct tumors (Guicciardi and Gores 2005).

In recent years, alternative types of programmed cell death to apoptosis have also been described (Sperandio et al. 2000). Paraptosis was first described in 2000 (Sperandio et al. 2000) and is a form of programmed cell death (PCD) biochemically and morphologically distinct from necrosis and apoptosis. Paraptosis is distinguished by the absence of apoptotic features, such as membrane blebbing, chromatin condensation, nuclear fragmentation, and cytoplasmic vacuolation, which are not influenced by caspase activation or inhibition. Growth factors, metallic complexes, natural compounds, and photodynamic therapy are numerous stimuli that can induce paraptosis (Al‐Madhagi 2024).

In recent years, increasing sensitivity to health, the inadequacy of synthetic drugs against growing diseases, and the detection of side effects have increased the tendency to use natural products (Çiftçi Yegin 2017). The traditional use of medicinal herbs implies a significant cultural and historical significance, appropriate for various foods available as traditional herbal medicines (Waheed et al. 2024). It is well‐known that some natural molecules in various plants effectively prevent human cancers. These cancer‐suppressive or preventive natural molecules have low toxicity and can reduce patients' pain during treatment (O'Connor 2015). However, the efficacy of these natural molecules in cancer treatment in plants is lower than that of synthetic drugs. However, different combinations of these molecules significantly increase the effectiveness of cancer treatment (Jiang et al. 2017; Wang et al. 2017). Therefore, it is crucial to discover appropriate combinations of various natural cancer suppressors in cancer treatment. When the current literature was examined, sumac, cumin (Goodarzi et al. 2020; Mahalakshmi et al. 2020), and thyme (Bozkurt et al. 2012; Desta et al. 2017) extracts exerted anticancer effects due to their antioxidant properties and polyphenol content. In addition, according to the existing literature, the impact of sumac extract on HCC has not been studied before, and the effects of thyme and cumin extracts on apoptosis and paraptosis and the synergistic effects of these three spices have not been investigated yet. Therefore, this study was planned to evaluate the impact of sumac, cumin, and thyme extracts on apoptosis and paraptosis in the HCC cell line.

## 2 Material and Methods

The study's experiments were conducted in the Erciyes University Genome and Stem Cell Centre (GENKÖK) Laboratory, and the spice extracts were prepared in the Laboratory of the Department of Pharmacognosy, Faculty of Pharmacy, Erciyes University.

### 2.1 Preparation of Sumac, Thyme, and Cumin Extracts

Sumac, thyme, and cumin spices were obtained commercially from the most preferred brand (Baghdad Baharat) since they are plants frequently preferred as spices. The powdered herbal material was extracted four times at room temperature by adding 70% methanol and mashed for 24 h each. The extracts were combined and concentrated in a rotavapor (37°C–38°C) under vacuum. All extracts were lyophilized and stored at −20°C until analysis. To prepare methanolic extract stocks, 4 mg of extract was dissolved in 20 μL of di‐methyl sulfoxide (DMSO) and 1980 μL of complete medium was pipetted and mixed with a vortex for complete homogenization. Fresh extract stocks were prepared before each experiment.

### 2.2 Cell Line and Reagents

The HepG2 cell line was used as a human liver HCC cell line in this study. The Erciyes University GENKÖK Centre lecturer provided the cell line. Reagents and chemicals were obtained from the relevant suppliers: Fetal bovine serum (FBS) (Biological Industries, USA), L‐glutamine (Pan Biotech, Germany), penicillin/streptomycin (Capricorn Scientific, Germany), Dulbecco's Modified Eagle's Medium (DMEM) (Sigma Aldrich, United States), MTT kit (Biotium, United States), 3,3'diaminobenzidine (DAB) solution (Thermo Fisher Scientific, United States), BCA Protein Assay kit (Thermo Fisher Scientific, USA) and antibodies (Bioss Antibodies, Woburn, MA, United States). All other reagents were obtained from Sigma (Sigma Aldrich, USA).

### 2.3 Cell Culture

High glucose complete DMEM medium containing 10% FBS, 1% L‐glutamine, and 1% penicillin/streptomycin was used as a growth medium. Cells were cultured at 37°C in an incubator with 5% CO_2_. All experiments were performed after the cells had adhered to the plate/flask overnight.

### 2.4 Cell Viability Test

HepG2 cells were seeded in 96‐well plates (1 × 10^5^ cells/plate) and left to adhere in the incubator for 24 h. Then, they were treated with sumac, cumin, and thyme extracts in single and combined doses determined from the literature (Goodarzi et al. 2020; Desta et al. 2017; Lee et al. 2018). One group was used as a control. Cells treated with single and combined doses were incubated for 24, 48, and 72 h, respectively, and the procedures were conducted according to the kit protocol. Then, the absorbance values in the wells were measured with a microplate reader (Promega, United States of America) at a 570 nm wavelength. IC_50_ values were calculated with the measured absorbance values. All experiments were repeated three times. According to the MTT test results, the viability of the cells did not decrease by up to 50% at 24 h, but since it significantly reduced at 72 h, all subsequent experiments were carried out based on 48 h.

### 2.5 Calculation of Combination Index

The combination index (CI) was calculated using the Chou‐Talay method to determine the synergistic or antagonistic effects of the extracts that were applied to HepG2 cells. CI was calculated with MTT absorbance values of single and combined doses. In the calculation, MTT absorbance values were entered into CompuSyn, and classification was performed according to the value on the *y*‐axis, with the fractional effect and CI graph drawn in the program. A synergistic effect means that combined doses are more cytotoxically effective than single doses; an additive effect means that the impact of single and combined doses are equal; and an antagonistic effect means that single doses are more cytotoxically effective than combined doses (Chou 2006).

### 2.6 Immunocytochemical Analyses

The expression levels of Bax, bcl 2, caspase‐3, −8, and −9 proteins were obtained by immunocytochemical staining analysis. HepG2 cells were seeded in 24‐well plates (0.24 × 10^5^ cells/well) and left to adhere in a CO_2_ incubator at 37°C for 24 h. The next day, the cells were treated with sumac, thyme, and cumin extracts at the determined (IC_50_ doses) single and combined doses for 48 h, and the cells were fixed with formic acid after 48 h of incubation. After 3% hydrogen peroxide (H_2_O_2_) was added and removed from the wells, each well was treated with serum block, and antibodies (Bax, bcl‐2, caspase‐3, −8, and −9) were added into the dilution buffer at a ratio of 1:400. The antibody‐dilution buffer mixture was added to the wells. Then, the plate was covered and kept overnight at +4°C. The next day, the plates were treated with a secondary antibody, streptavidin peroxidase was added to each well, and we waited for 20 min. Then, the DAB solution was diluted at a ratio of 80:1000 and added to each well. After brown staining was observed in the wells, the nuclei of the cells were stained with Gill hematoxylin stain for 30 s. Cells were kept at +4°C with distilled water until imaging was performed. The wells were photographed at 40× magnification, and the intensity of antibodies was measured in the Image J program.

### 2.7 Western Blot Analysis

Expression levels of beta‐actin and mTOR proteins were evaluated using Western Blot analysis. To perform Western Blot analysis, HepG2 cells were seeded in 25 cm^2^ flasks and treated with sumac, thyme, and cumin extracts at determined doses for 48 h. RIPA lysis solution was added to lysed cells, and the protein concentrations of cell lysates were determined using the BCA Protein Assay kit. When the curve of the standard solutions was 99%, the measurements were considered accurate. Calculations were made according to the concentration of the samples, and appropriate amounts of distilled water and loading buffer were added, thus preparing for the total amounts to be loaded on the gel. Proteins were heated in a heater at 95°C for 5 min.

The prepared samples and marker were loaded onto the prepared gel in 20 μL, analyzed by 4%–20% gradient sodium dodecyl sulfate (SDS)‐polyacrylamide gel electrophoresis for protein separation and electrotransferred onto polyvinylidene difluoride (PVDF) membranes. Β‐actin was used to ensure the accuracy of the loading. Proteins were transferred to the membrane, blocked with 5% (w/v) skimmed milk, and washed with Tris‐buffered saline‐Tween solution (TBST). The membrane was probed with a specific primary antibody (mTOR, 1:1000; beta‐actin, 1:1000) overnight at 4°C. The next day, the membrane was washed with TBS‐T 6 times for 5 min each and kept at 37°C for 2 h with the secondary antibody (HRP‐conjugated Secondary Antibody, 1:1000). After washing with TBS‐T 6 times for 5 min each, it was prepared for imaging. All antibodies were diluted in TBS‐T containing 5% dry milk. Before probing a new antibody, the membrane was treated with strip a buffer for 10 min to remove the previous antibody from the membrane. For imaging, 750 μL each of ECL A and B solutions were added to the membrane, and images were taken on a ChemiDOC (BioRad, United States of America) imager. The device software measured and analyzed the protein bands obtained from the recorded images.

### 2.8 Paraptosis Tests

ER stress was measured to determine the extracts' effects on paraptosis. Changes in the expression levels of GRP‐78/BIP, an indicator of ER stress, and DDIT3/CHOP, a transcription factor, were determined by immunocytochemistry staining analysis according to the protocol described above.

### 2.9 Statistical Analyses

SPSS 22.0 package program, Microsoft Excel 2019, GraphPad Prism 9, and CompuSyn program were used for statistical data analysis. Differences between groups were analyzed by one‐way analysis of variance (ANOVA). The Bonferroni test was used to compare the data multiple times. *p* < 0.05 was considered statistically significant.

## 3 Results

### 3.1 Cell Viability

**FIGURE 1:**
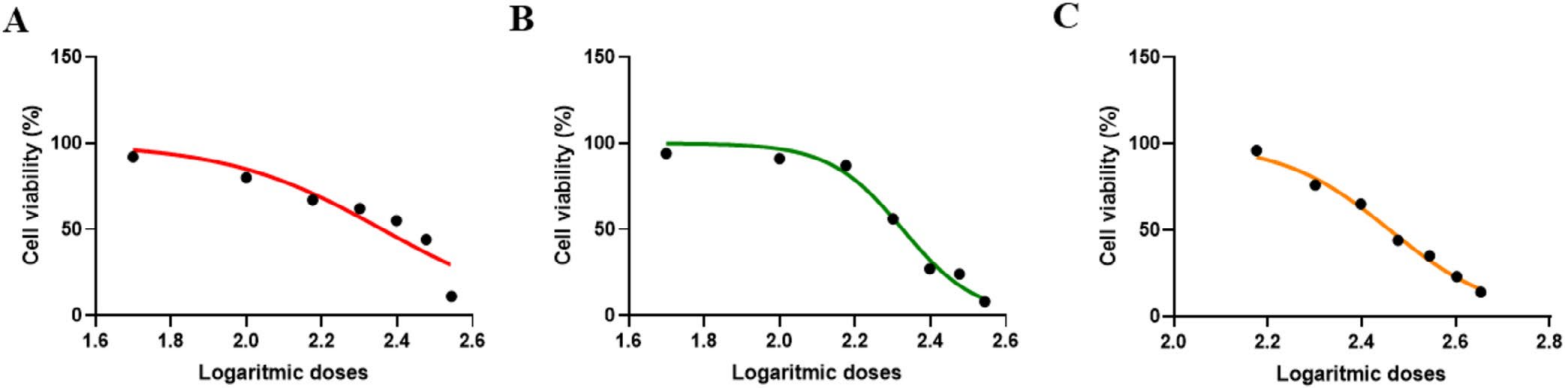
IC_50_ graphs of sumac (A), thyme (B) and cumin (C) extracts applied to HepG2 cells for 48 h according to MTT assay.

For the MTT test, 50, 100, 150, 150, 150, 200, 200, 250, 250, 300, and 350 μg/mL doses of sumac and thyme extracts and 150, 200, 250, 300, 350, 400, and 450 μg/mL doses for cumin were applied. The double and triple combined doses used in the MTT test are shown in Table 1. Synergistic and antagonistic effects of the spices were determined by calculating the CI of the combined doses with the MTT results. IC_50_ values could not be calculated since all extracts showed a proliferative effect with 24 h of application and did not show a toxic effect. Since cell viability decreased below 50% in the 72 h application, IC_50_ values of only 48 h were calculated. In the other subsequent applications, 48 h application was continued based on the 48 h application. The IC_50_ values of sumac, thyme, and cumin extracts were 229.2 μg/mL (Figure 1A), 211.9 μg/mL (Figure 1B) and 288.1 μg/mL (Figure 1C), respectively, with 48 h of extract application by MTT test.

**TABLE 1:** MTT test double and triple combined doses.

Combinations	Sumac (μg/mL)	Thyme (μg/mL)	Cumin (μg/mL)	Total (μg/mL)
**Sumac‐ Thyme (S‐T)**				
1	125	100	—	225
2	125	50	—	175
3	62.5	100	—	162.5
4	62.5	50	—	112.5
**Thyme‐ Cumin (T‐C)**				
1	—	100	150	250
2	—	100	75	175
3	—	50	150	200
4	—	50	75	125
**Sumac‐Cumin (S‐C)**				
1	125	—	150	275
2	125	—	75	200
3	62.5	—	150	212.5
4	62.5	—	75	137.5
**Sumac‐Thyme‐Cumin (S‐T‐C)**				
1	27.8	66.7	33.3	127.8
2	27.8	22.2	100	150
3	27.8	22.2	33.3	83.3

### 3.2 Synergistic/Antagonistic/Additive Effect

**FIGURE 2:**
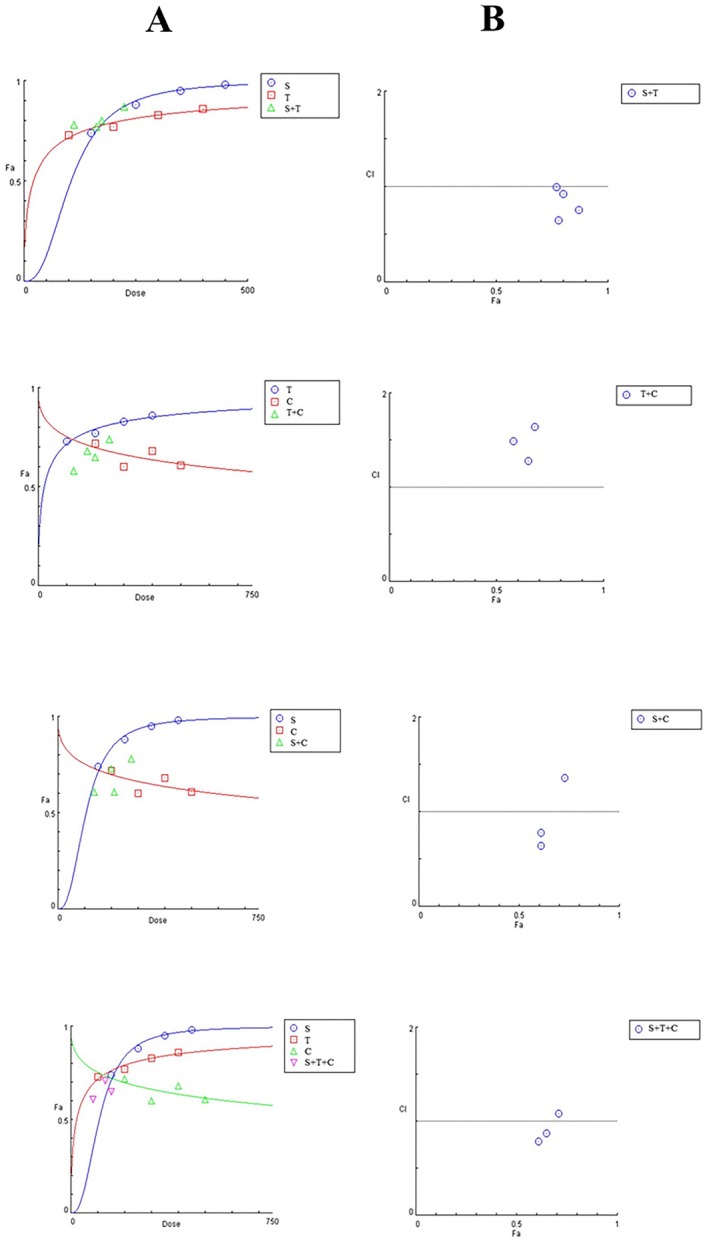
Dose‐effect and combination index graphs, A: Dose‐effect graphs, B: Combination index graphs, S: Sumac, T: Thyme, C: Cumin.

As a result, the CI value for sumac‐thyme was 0.92, that is, additive effect. This finding indicates that single and double doses of sumac and thyme extracts have the same effect. The CI value for thyme‐cumin was 1.64. In other words, thyme and cumin extracts show an antagonist effect. Single‐dose applications of thyme and cumin extracts were more effective than double‐dose applications. The CI value for sumac‐cumin was 0.73, and sumac and cumin extracts showed a synergistic effect. Finally, the CI value for sumac‐thyme‐cumin was 0.65. In other words, the triple dose showed a synergistic effect and was more effective than single‐dose applications (Figure 2).

### 3.3 Immunocytochemistry Analysis

**FIGURE 3:**
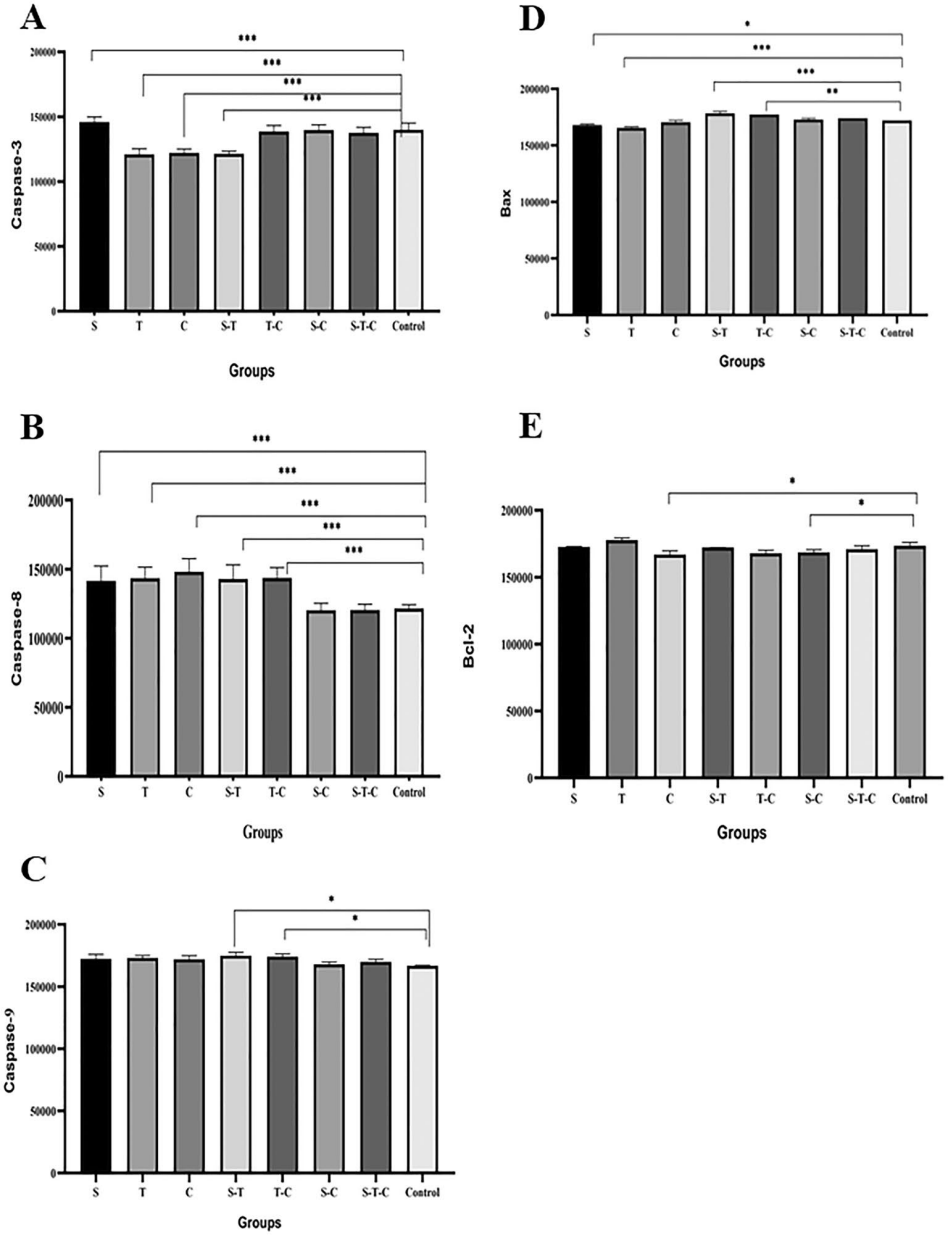
Caspase‐3 (A), caspase‐8 (B), caspase‐9 (C), Bax (D), and bcl‐2 (E) protein expression levels in HepG2 cells treated with sumac, thyme, and cumin extracts for 48 h. One‐way ANOVA test was used to analyze the data. The Tukey test was applied for post hoc analyses. For single doses, sumac 250 μg/mL, thyme 200 μg/mL, and cumin 300 μg/mL. For double combined doses, (1) 125 μg/mL sumac and 50 μg/mL thyme, (2) 100 μg/mL thyme and 75 μg/mL cumin, (3) 125 μg/mL sumac and 75 μg/mL cumin. For triple combined doses, 27.8 μg/mL sumac, 22.2 μg/mL thyme, 100 μg/mL cumin.

The caspase‐3 protein expression level in the S group was significantly higher than in all groups (*p* < 0.0001). When caspase‐3 protein expression levels were compared with the control group, they were significantly higher in the S group and significantly lower in the T, C, and S‐T groups (*p* < 0.0001) (Figure 3A). Caspase‐8 protein expression level was higher in the C, S‐C, and S‐T groups compared to other groups (*p* < 0.0001). When compared with the control group, caspase‐8 protein expression levels were significantly higher in the S, T, C, S‐T, and T‐C groups (*p* < 0.0001) (Figure 3B). Caspase‐9 protein expression levels were significantly higher in the S‐T and T‐C groups (*p* < 0.05). When compared with the control group, only S‐T (*p* = 0.013) and T‐C (*p* = 0.0189) groups had significantly higher protein expression levels (Figure 3C). Bax protein expression level was significantly higher in the S‐T and T‐C groups (*p* < 0.05). Bax protein expression level was significantly higher in the T‐C group than in the S‐C and control groups (*p* < 0.01). Bax protein expression levels were significantly lower in the S and T groups compared to the control group. In contrast, they were significantly higher in the S‐T and T‐C groups (*p* < 0.05) (Figure 3D). Bcl‐2 protein expression level was significantly higher in the T group compared to C, T‐C, S‐C, and S‐T‐C groups (*p* < 0.01). When compared with the control group, it was significantly lower only in the C (*p* = 0.0327) and S‐C (*p* = 0.0215) groups (Figure 3E).

### 3.4 Western Blot Analysis

**FIGURE 4:**
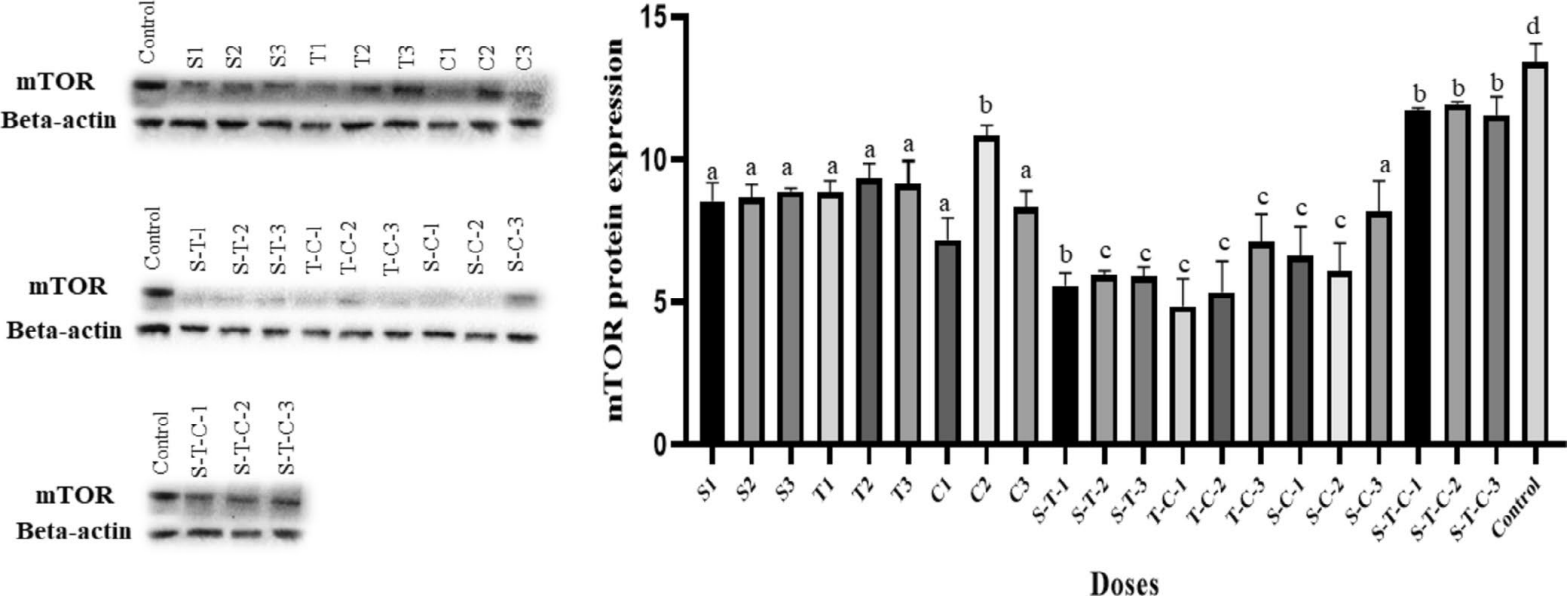
A Western blot image and graph of the expression level of mTOR protein in HepG2 cells treated with sumac, thyme, and cumin extracts for 48 h are shown. A one‐way ANOVA test was used to analyze the data. The Tukey test was applied for post hoc analyses. There is a statistically significant difference between the groups expressed with different exponents in multiple comparisons, *p* < 0.05.

When the mTOR protein expression levels of the groups were analyzed, a significant difference was observed between all groups in multiple comparisons (*p* < 0.0001). The mTOR protein expression level was significantly higher in the control group compared to all groups (*p* < 0.0001). The mTOR protein expression level was lower in the S‐T, T‐C, and S‐C groups, which were double combined doses, compared to the other groups (*p* < 0.0001). The lowest mTOR protein expression level was observed in the T‐C‐1 group (*p* < 0.0001). When single doses were compared among themselves, except for C2, the mTOR expression level was lower in both groups compared to the sumac and thyme groups (*p* < 0.0001). In triple combined doses, the mTOR protein expression level was higher than in single and double combined doses (*p* < 0.0001) (Figure 4).

### 3.5 Paraptosis

**FIGURE 5:**
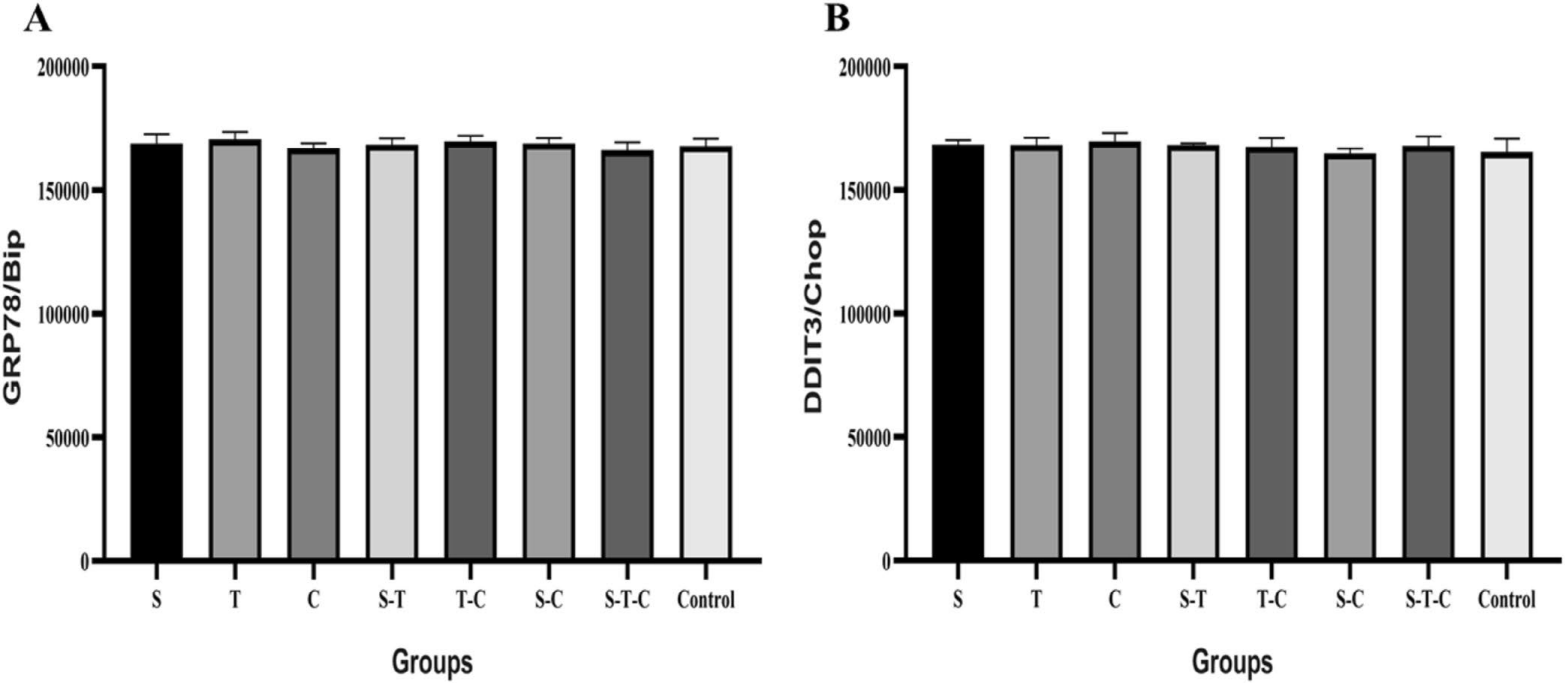
GRP78/Bip (A) and DDIT3/Chop (B) protein expression levels in HepG2 cells treated with sumac, thyme, and cumin extracts for 48 h. For the experiments, cells were seeded in 24‐well plates at 0.24 × 10^5^ cells/well, and all experiments were repeated 3 times. A one‐way ANOVA test was used to analyze the data. The Tukey test was applied for post hoc analyses. S1, S2, and S3 were 150, 250, and 350 μg/mL sumac, respectively; T1, T2, and T3 were 100, 200, and 300 μg/mL thyme, respectively; C1, C2, and C3 were 200, 300, and 400 μg/mL cumin, respectively; S‐T‐1 was 125 μg/mL sumac and 100 μg/mL thyme, S‐T‐2 was 125 μg/mL sumac and 50 μg/mL thyme, S‐T‐3 was 62.5 μg/mL sumac and 100 μg/mL thyme, T‐C‐1 was 100 μg/mL thyme and 150 μg/mL cumin, T‐C‐2 was 100 μg/mL thyme and 75 μg/mL cumin, T‐C‐3 was 50 μg/mL thyme and 150 μg/mL cumin, S‐C‐1 was 125 μg/mL sumac and 150 μg/mL cumin, S‐C‐2 was 125 μg/mL sumac and 75 μg/mL cumin, S‐C‐3 was 62.5 μg/mL sumac and 150 μg/mL cumin, S‐T‐C‐1 was 27.8 μg/mL sumac, 66.7 μg/mL thyme, 33.3 μg/mL cumin, S‐T‐C‐2 was 27.8 μg/mL sumac, 22.2 μg/mL thyme, 100 μg/mL cumin, and S‐T‐C‐3 was 27.8 μg/mL sumac, 22.2 μg/mL thyme, 33.3 μg/mL cumin.

GRP78/Bip and DDIT3/Chop protein expression levels of all groups were similar, and no significant difference was observed between the groups (*p* > 0.05) (Figure 5).

## 4 Discussion

This study investigated the effects of sumac, cumin, and thyme methanolic extracts on apoptosis and paraptosis in HepG2 cells, an HCC cell line. When the literature was examined, no study examined the effect of sumac, thyme, and cumin extracts on apoptosis and paraptosis in HepG2 cells. At the same time, the synergistic effects of these spices have not been investigated. Therefore, this study is the first in the literature.

Vegetables, spices, and fruits contain natural compounds that suppress cancer‐forming mechanisms and activate disease‐preventing mechanisms. Such components stimulate antioxidant, anti‐proliferative, anti‐tumor, and anti‐inflammatory mechanisms involved in cancer formation. Some components show cytotoxic effects on cancer cells and selectivity for non‐cancerous cells to be unaffected (Mandlik and Mandlik 2022). Many plants and plant components are used worldwide to treat chronic liver diseases due to budget efficiency, high safety margins, long‐lasting benefits, and fewer side effects. Herbal medicines suppress the development of several types of cancer by blocking cell growth at various stages of the cell cycle, inhibiting signaling pathways, and gene expression. Primarily, herbal drugs exert their anticancer effects through autophagic and apoptotic pathways (Mandlik and Mandlik 2021). However, such products have limitations regarding dose control, bioavailability, and efficacy (Ahmed et al. 2021).

The sumac phytochemicals play a role in cell cycle arrest, induce apoptosis and autophagy, reduce DNA damage, and inhibit angiogenesis, mutant tumor suppressors, and metastasis, among other mechanisms contributing to its anti‐cancer effects (Alsamri et al. 2021). In studies conducted with aqueous and alcohol extracts of the sumac plant in various cell lines (MCF‐7, MDA‐MB‐231, T47D, Caco‐2, HT‐29), sumac extract decreased cell viability in a dose‐ and time‐dependent manner (El Hasasna et al. 2015; Athamneh et al. 2017). In contrast to these studies, in a study investigating the anticancer effects of sumac hydroalcoholic extract in MCF‐7, PC‐3, and SKOV3 cell lines, sumac extract did not show a cytotoxic effect at applied doses of 0–100 μg/mL (Gabr and Alghadir 2021). At the same time, in a study evaluating the antioxidant effects of sumac ethanolic extract in BV‐2 cells, no cytotoxic effect was observed at 24 and 48 h (Khalil et al. 2021). In the present study, similar to these studies, no cytotoxic effect of sumac methanolic extract doses applied to HepG2 cells was observed at 24 h, while IC_50_ value was 229.2 μg/mL at 48 h. The IC_50_ value was not calculated, since the percentage of viable cells dropped below 50 at 72 h. In other words, a time‐dependent decrease in cytotoxic dose was observed in the current study.

In various studies on the effects of aqueous and alcohol extracts of thyme on HepG2 cells were investigated, dose‐dependent cytotoxic effects were observed at 24 and 48 h (Kozics et al. 2013; Taghouti et al. 2020). In the present study, the IC_50_ value of 48 h methanolic thyme extract was 211.9 μg/mL in HepG2 cells, with a dose close to that of Taghouti et al. (2020). However, unlike them, no cytotoxic effect was observed in this study with 24 h application. There are also studies on non‐alcoholic oily extracts of thyme In these studies, very different doses were applied, and IC_50_ doses were determined differently (Al‐Menhali et al. 2015; Niksic et al. 2021; Sertel et al. 2011). The extract prepared by dissolving the plants may show activity at varying doses in the same cell line, and the same type of extract may show activity at different doses in different cells.

In a study planned by Prakash and Gupta (2014) to evaluate the cytotoxic activity of cumin ethanolic extract, 100 μg/mL cumin ethanolic extract was applied to seven cancer cell lines (Colon 502713, Colo‐205, Hep‐2, A‐549, OVCAR‐5, PC‐5, SF‐295) for 48 h. Significant decreases in cell viability were detected (Prakash and Gupta 2014). A study with 0.1 μL/mL cumin essential oil inhibited Hela cells by 79% (Allahghadri et al. 2010). In contrast to these studies, El‐Dawy et al. (El‐Dawy et al. 2019) evaluated the cytotoxic effect of cumin at concentrations of 0–100 μg/mL in four different cell lines (HepG2, HCT‐116, MCF‐7, PC‐3). They found no cytotoxic effect in other cell lines except the PC‐3 cell line. In PC‐3 cells, the IC50 value was found to be 24.5 μg/mL. In the present study, an IC_50_ dose of 288.1 μg/mL was determined by application of 48 h methanolic cumin extract to HepG2 cells. The study's results were higher than the study in which alcohol extract was applied. However, since that study was performed in a different cell line, it is not unexpected that the results were different. As can be seen, diverse types of extracts of sumac, thyme, and cumin spices showed different effects on various kinds of cell lines. The reason for these differences may be related to the sensitivity of the cell lines, the aqueous/ethanolic/methanolic nature of the extracts affecting the effectiveness of the extracts, or the differences in the places where the spices are grown, the time of collection, processing, or storage may change the bioactive component content of the spices.

Single and combined herbal preparations have been used extensively throughout history to treat various diseases (Al‐Bayati 2008). The combination of different plant extracts may show physiologically different effects compared to their single use, depending on the difference between the various phytochemicals in their structure. This may result in synergistic, antagonistic, or additive effects. This effect refers to the impact of single or combined use on physiological health potential. In synergistic interactions, the combined use of physiological health effects is higher than that of the single use. In antagonistic interactions, on the contrary, the physiological potential of combined use is less than single use. In additive interaction, there is no difference between single and combined use regarding physiological health potential (Wang et al. 2015). Although there are no studies in the literature in which double or triple doses of these three spices are used and their synergistic effects are examined, there are studies conducted with different extracts of these spices with various plants.

Antioxidant capacity and inhibitory effect of single and combined use of sumac and raspberry extracts in normal and cancerous cells were investigated. RAT‐2, C2C12, MCF‐10A, MCF‐7, and 4D/WT were used as cell lines. Remarkably, the combination of sumac and raspberry showed maximum inhibition in the growth of both rat colon and human breast cancer cells, with relatively low cytotoxicity against normal rat colon and human breast epithelial cells (Wang et al. 2015). In a study investigating the antiproliferative effect of single and combined use of thyme and sage essential oils, Alexa et al. (Alexa et al. 2018) applied thyme and sage essential oils at concentrations of 50 μg/mL and 100 μg/mL to A375 and B164A5 cell lines. They evaluated the cytotoxic effect of single and combined use by MTT assay. In both cell lines, sage essential oil was more effective in inhibiting proliferation. The antiproliferative effect increased linearly with the combined use of essential oils.

In other words, thyme and sage essential oils showed a synergistic effect (Alexa et al. 2018). In a study evaluating the antibacterial and antioxidant effects of the combination of cumin and coriander essential oil, different concentrations of cumin and coriander seed combination were applied to the CCD‐18Co cell line. According to MTT results, the coriander/caraway seed oil combination showed no cytotoxic effect on the human regular colon cell line. The coriander/caraway seed oil combination showed synergistic interactions in antibacterial and antioxidant activities (Bag and Chattopadhyay 2015). Similar to the above studies, some combinations were synergistic in this study. The sumac and thyme duo was additive, the thyme and cumin duo was antagonistic, the sumac and cumin duo was synergistic, and the sumac, thyme, and cumin trio was synergistic. In other words, dual and single use of sumac and thyme extracts had no difference in cell viability. Single use of thyme and cumin extracts caused a more significant reduction in cell viability than dual use. In sumac and cumin extracts, dual use caused a greater decrease in cell viability. In triple use, cell viability decreased more than in single use. The higher synergism between sumac and cumin may explain the synergistic effect of triple use. This higher synergism may have had a dominant effect on the antagonism between thyme and cumin.

The ratio of anti‐apoptotic proteins to pro‐apoptotic proteins determines whether a cell will enter apoptosis. In response to death signals, the caspase cascade is activated to carry out the apoptotic process (Cheung et al. 2002).

Studies have evaluated the effect of various sumac, thyme, and cumin extracts on apoptosis in different cell lines. Studies have shown that sumac extract caused overexpression of caspase‐3 and a decrease in bcl‐2 (Gabr and Alghadir 2021), and increased the levels of apoptosis‐related factors such as Bax/bcl‐2 ratio and cleaved caspase‐3 and ‐9 activity (Kim et al. 2019), thus activating the apoptotic process and significantly inhibiting cancer cell growth, proliferation, and viability. The expression levels of caspase‐3 and ‐7 proteins increased in a concentration‐dependent manner in colon and breast cancer cells treated with various concentrations of thyme aqueous (Al‐Menhali et al. 2015) and oily (Kubatka et al. 2019) extracts. In a study investigating the anticancer activity of cumin extract in the SW480 CC cell line, cumin extract significantly decreased the proliferation and viability of the SW480 CC cell line depending on the concentration time. In addition, the expression of Bax and bcl‐2 genes increased and decreased in SW480 cells treated with cumin extract (Rostami et al. 2022).

This study measured expression levels of Bax, bcl‐2, caspase‐3, −8, and −9 proteins to evaluate the effect of sumac, thyme, and cumin extracts on apoptosis‐related proteins in HepG2 cells. When the effects of sumac, thyme, and cumin extracts applied to HepG2 cells for 48 h on Bax and bcl‐2 protein expression levels were examined, a pro‐apoptotic protein, Bax, protein expression level was higher in T‐C and S‐T groups. In comparison, an anti‐apoptotic protein, bcl‐2, protein expression level was lower in C, T‐C, and S‐C groups. In other words, the Bax/bcl‐2 ratio was higher in extracts using thyme and cumin. According to the CI values obtained from the MTT test results, thyme and cumin extracts showed an antagonistic effect on cell viability. However, when Bax and bcl‐2 protein expression levels were analyzed, thyme and cumin extracts showed a synergistic effect for these proteins and caused an increase in apoptosis. When the expression levels of apoptotic sumac proteins, caspase‐3, −8, and −9 proteins were examined, caspase‐3 protein expression level was the highest in the S group, high in S‐C, T‐C, and S‐T‐C groups and the lowest in the S‐T group. In other words, caspase‐3 protein was expressed more in the sumac groups. Caspase‐3 expression was low in the S‐T group but high in the S‐C, T‐C, and S‐T‐C groups. In other words, it is possible to say that the effect of cumin on caspase‐3 protein is greater than that of thyme. Considering that caspase‐3 protein expression was also the highest in the S group, this supports the additive effect between sumac and thyme and the synergistic effect between sumac and cumin obtained from the CI values. When the expression levels of caspase‐8 protein were analyzed, they were higher in the T, C, and T‐C groups. Although thyme and cumin showed antagonistic effects on cell viability, they showed synergistic effects on caspase‐8 protein expression. Finally, caspase‐9 protein expression was higher in the S‐T, T‐C, and T groups. Despite the additive effect between sumac and thyme and the antagonistic effect between thyme and cumin, caspase‐9 protein expression was higher in the groups in which they were used together. In general, the sumac plant mainly affected caspase‐3 protein expression, the thyme plant mostly affected caspase‐8, −9, Bax, and bcl‐2 protein expression, and the cumin plant mainly affected caspase‐8, Bax, and bcl‐2 protein expression. The fact that thyme and cumin extracts affected more proteins in the apoptotic pathway than sumac extract supports the finding that thyme and cumin extracts were more effective than sumac extract. The fact that the additive, synergistic, and antagonistic effects of the extracts on cell viability do not match their effects on protein expression levels can be explained by the fact that their effects on proliferative and anti‐proliferative proteins other than these proteins may differ. Such different results may have been obtained due to effects on other cellular death pathways such as autophagy.

Several pathways and processes have been involved in HCC progression. One of them, Ras/Raf/MAPK and PI3K/AKT–mTOR pathways, is frequently activated in HCC (Forner et al. 2018). mTOR plays a critical role in regulating the balance between cell proliferation and autophagy in response to cellular stress in cancer cells (Yang et al. 2018). In a study investigating the anti‐leukemic potential of sumac extract against THP‐1 cells, sumac extract controlled THP‐1 cell proliferation and apoptosis by regulating the PI3K‐Akt–mTOR signaling pathway (Tlili et al. 2021). In a study evaluating the effect of cumin aqueous extract on chromium‐induced oxidative damage in WRL‐68 cells, cumin extract reversed the effect of chromium on reducing mTOR expression in cells, restored it to normal levels, and reduced the degree of autophagic cell death (Mahalakshmi et al. 2020). This study applied Western blot analysis to evaluate the effect of sumac, thyme, and cumin extracts on mTOR protein expression levels in the HepG2 cell line. Inhibition of mTOR, an indicator of apoptosis, was higher in the S‐T, T‐C, and S‐C groups. mTOR inhibition was highest in the T‐C group. This supports the finding that thyme and cumin extracts cause more apoptosis, as shown in other findings.

Paraptosis is a vital cell death mechanism that produces anti‐tumor effects in various cancer subtypes. Many natural and synthetic compounds have been identified as potential targets to induce paraptosis in different cancer cell lines (Fontana et al. 2020). The effects of other compounds on paraptosis have been examined in the literature. However, the effects of sumac, thyme, and cumin extracts on paraptosis have not been examined. Yoon et al. (Yoon et al. 2010) showed that curcumin could induce paraptosis in different breast cancer cell lines by promoting mitochondrial and ER swelling and vacuolization resulting from their combination. In another study, ginsenoside induced both caspase‐dependent apoptosis and caspase‐independent paraptosis in HCT116 and SW480 cells (Li et al. 2011). In a study evaluating the effect of hesperidin on paraptosis in HepG2 cells, hesperidin induces paraptosis (Yumnam et al. 2016). In this study, GRP78/Bip and DDIT3/Chop protein expression levels were measured by immunocytochemistry staining analysis to evaluate the paraptosis effect of the extracts in HepG2 cells. As a result of the study, no significant difference was observed between the extract‐treated and untreated groups. Therefore, sumac, thyme, and cumin extracts did not significantly affect paraptosis. These extracts may show their anti‐tumor effect on the HepG2 cell line through apoptosis or other cell death pathways.

This research has limitations; it determined only the cytotoxic doses of sumac, thyme, and cumin in HepG2 cells. It is necessary to analyze the cytotoxic effect levels of these extracts on normal, healthy liver cells to say that sumac, cumin, and thyme extracts can be used in HCC in humans and are beneficial. However, studies have shown that sumac, cumin, and thyme do not show cytotoxic effects on healthy cells or do not have less cytotoxicity than cancer cells (Mohamed et al. 2022; Mirian et al. 2015; Benedetti et al. 2023; Scazzocchio et al. 2016). In addition, since the study budget was insufficient to buy a new cell line and we did not have a healthy cell line, this constitutes the limitation of the study.

This study evaluated the effects of single, double, and triple combined doses of sumac, thyme, and cumin methanolic extracts on apoptosis and paraptosis in the HepG2 cell line. This is the first since there is no study that exists in the literature. The study shows that using sumac, thyme, and cumin plants may have a supportive effect preventing and treating cancer. However, due to the many health problems that may be caused by the unconscious use of herbal products, extensive animal and human studies are needed to recommend the routine use of these products to patients. It is necessary to determine the safe and effective doses for cancer patients, especially sensitive groups, with comprehensive studies to be conducted. Since these patients receive diverse types of drug therapy, the issue of whether the herbal products used will interact with drugs should be investigated in detail. In addition, the contents of many spice mixtures sold in the market claiming to be effective against many diseases should be analyzed, and the synergistic/antagonistic effects of the spices in these mixtures with each other should be determined. The activities of different bioactive components in these different spices can be increased by determining these effects.

## Author Contributions

**Yagmur Yasar Firat:** conceptualization (lead), data curation (equal), formal analysis (equal), funding acquisition (equal), investigation (equal), methodology (equal), software (equal), validation (equal), visualization (equal), writing – original draft (equal), writing – review and editing (equal). **Betul Cicek:** conceptualization (equal), funding acquisition (equal), project administration (lead), supervision (lead), writing – review and editing (equal). **Ayca Kara:** conceptualization (equal), data curation (equal), formal analysis (equal), investigation (equal), methodology (equal), software (equal), visualization (equal), writing – review and editing (equal). **Nurefsan Konyaligil Ozturk:** conceptualization (equal), data curation (equal), formal analysis (equal), investigation (equal), visualization (equal), writing – review and editing (equal). **Selen Ilgun:** methodology (equal), visualization (equal), writing – review and editing (equal).

## Conflicts of Interest

The authors declare no conflicts of interest.

## Data Availability

The data that support the findings of this study are available on request from the corresponding author.
